# The Biological Activities of Cinnamon, Geranium and Lavender Essential Oils

**DOI:** 10.3390/molecules191220929

**Published:** 2014-12-12

**Authors:** Monika Sienkiewicz, Anna Głowacka, Edward Kowalczyk, Anna Wiktorowska-Owczarek, Marta Jóźwiak-Bębenista, Monika Łysakowska

**Affiliations:** 1 Environmental Biology Department, Medical University of Lodz, ul Żeligowskiego 7/9, Lodz 90-752, Poland; E-Mail: anna.glowacka1@umed.lodz.pl; 2 Pharmacology and Toxicology Department, Medical University of Lodz, ul Żeligowskiego 7/9, Lodz 90-752, Poland; E-Mails: edward.kowalczyk@umed.lodz.pl (E.K.); anna.wiktorowska-owczarek@umed.lodz.pl (A.W.-O.); marta.jozwiak-bebenista@umed.lodz.pl (M.J.-B.); 3 Department of Microbiology and Medical Laboratory Immunology, Medical University of Lodz, ul Pomorska 251, Lodz 92-213, Poland; E-Mail: monika.lysakowska@umed.lodz.pl

**Keywords:** *Acinetobacter* sp., cinnamon oil, ESBL-positive strains, geranium oil, Minimal Inhibitory Concentration, lavender oil

## Abstract

*Acinetobacter* sp. represent an important cause of nosocomial infections. Their resistance to some antibiotics, their ability to survive on inanimate surfaces in the hospital environment and their ability to produce biofilms contributes to their virulence. The aim of the study was to determine the antibacterial properties of cinnamon, lavender and geranium essential oils against bacteria of the genus *Acinetobacter* isolated from several clinical materials and from the hospital environment. A comprehensive evaluation of the susceptibility of *Acinetobacter* sp. clinical strains to recommended antibiotics was performed. The constituents of cinnamon, lavender and geranium essential oils were identified by GC-FID-MS analysis, and their Minimal Inhibitory Concentrations (MICs) against tested clinical strains were determined by the micro-dilution broth method. In addition, the effects of essential oils on the viability of human microvascular endothelial cells (HMEC-1) and glioblastoma cell line (T98G) were evaluated. Cinnamon bark oil was the most active against clinical and environmental strains of *Acinetobacter baumannii* with MIC values ranging from 0.5 to 2.5 µL/mL. The MIC values for geranium oil were between 7.5 and 9.5 µL/mL, and between 10.5 and 13.0 µL/mL for lavender oil. These essential oils can be best employed in the fight against infections caused by bacteria from *Acinetobacter* genus as components of formulations for hygiene and disinfection of hospital environment.

## 1. Introduction

One of the most common causes of difficult to treat infection is the increasingly multidrug resistant Gram-negative bacillus *Acinetobacter baumannii*. The species has been observed in diverse health care infections, including bacteremia, pneumonia, meningitis, urinary tract, and wound infections. The key factors in the spread of strains in the hospital environment are the growth of resistance to antibiotics and tolerance of desiccation. As therapeutic options for multidrug resistant *Acinetobacter* infections are limited, the development or discovery of new therapies is very important [1,2,3]. Essential oils offer the chance not only to fight infection, but also inhibit the growth of microbial resistance. Essential oils with various chemical compositions were chosen for this study: cinnamon bark oil with cinnamaldehyde as a main constituent, geranium oil with citronellol and geraniol, and lavender oil with linalool and linalyl acetate. According to Inouye *et al*., cinnamon bark oil and its major components exert antibacterial effects on major respiratory pathogens such as *Haemophilus influenzae*, *Streptococcus pneumoniae* and *Streptococcus pyogenes* [4,5]. Sivamani and Sahul Hameed recommended cinnamon and geranium oils against bacteria such as *Staphylococcus aureus*, *Streptococcus pneumoniae*, *Salmonella typhi*, *Escherichia coli*, *Pseudomonas aeruginosa*, *Shigella dysenteriae*, and *Proteus mirabilis* isolated from HIV-positive patients [6]. Probuseenivasan *et al*. report that cinnamon and geranium oils inhibitGram-positive and Gram-negative strains such as *Escherichia coli* ATCC 25922, *Klebsiella pneumoniae* ATCC 15380, *Pseudomonas aeruginosa* ATCC 27853 and *Proteus vulgaris* MTCC 1771 [7]. Lavender oil possesses not only antibacterial and antifungal activity, but is also known to have astringent and anti-inflammatory properties, as well as accelerate wound healing and reduce scarring [8,9]. A great advantage of essential oils is that their use not associated with long-term genotoxic risk. Some of them show an antimutagenic activity that could well be linked to an anticarcinogenic activity [10]. The pro-oxidant activity of essential oils or some of their constituents is very efficient in reducing local tumor volume or tumor cell proliferation by apoptotic and necrotic effects [11]. However, some of their constituents may be considered secondary carcinogens after metabolic activation [12]. Cinnamaldehyde, carvacrol, carvone and thymol have no significant toxic effects *in vivo* whilst *in vitro* they exhibit mild toxic effects at the cellular level [13]. According to Lahlou *et al.*, intravenous injection of the monoterpene alcohol terpinen-4-ol decreased mean aortic blood pressure in a dose-related manner, in conscious DOCA-salt hypertensive rats [14]. Then, essential oils obtained mainly from *Apiaceae*, *Rutaceae*, *Polygonaceae*, and *Hypericaceae* family have phototoxic activity [15]. The cases reports described by Bleasel *et al*., highlight the importance of allergic contact dermatitis associated with essential oil exposure in an occupational setting. However the risk of sensitization mainly concerns employees within the aromatherapy and massage industry [16].

## 2. Results and Discussion

### 2.1. Chemical Composition of the Cinnamon, Geranium and Lavender Essential Oils

Chemical analysis of the cinnamon essential oil revealed the presence of three main constituents: cinnamaldehyde (76.8%), methoxycinnamaldehyde (11.7%) and cinnamyl acetate (3.2%). According to the requirements of the European Pharmacopoeia (Sixth Edition) and the Polish Pharmacopoeia VIII, six of the nine main components of the tested oil were determined [17,18]. Geranium oil was found to include mainly citronellol (26.7%) and geraniol (13.4%), followed by nerol (8.7%), citronellyl formate (7.1%), isomenthone (6.3%), linalool (5.2%), 10-epi-γ-eudesmol (4.4%) and geranyl formate (2.5%). The composition of the tested geranium oil was consistent with the ISO-4731 standard. The analysis of the tested lavender oil showed that its composition met the requirements of the European Farmacopoeia (Sixth Edition) and the Polish Farmacopoeia VIII. The two main constituents of the lavender oil were found to be linalool (34.1%) and linalyl acetate (33.3%), with other significant components being ocymene and lavandulil acetate (3.2% each).

### 2.2. Susceptibility Testing of Clinical Acinetobacter Baumannii Strains to Antibiotics

**Figure 1:**
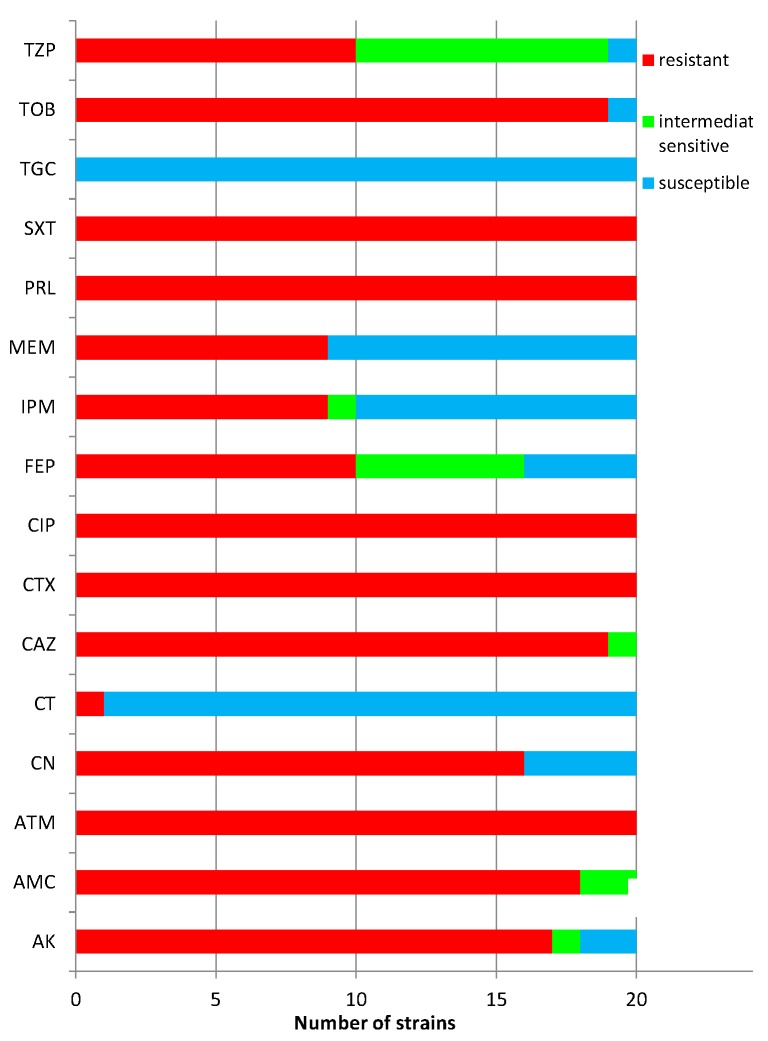
The susceptibility of the *Acinetobacter baumannii* strains to antibiotics.

All tested *Acinetobacter baumannii* strains isolated from patients, hospital equipment and from the environment were resistant to antibiotics: monobactam-ATM, cephalosporin-CTX, quinolon-CIP, β-lactam-PLR and SXT recommended for susceptibility testing. All of the tested strains were resistant to TGC and only one was resistant to CT. The results of susceptibility testing to antibiotics are presented in Figure 1.

### 2.3. The Susceptibility of Acinetobacter Baumannii Bacterial Strains to Cinnamon Bark Oil

**Figure 2:**
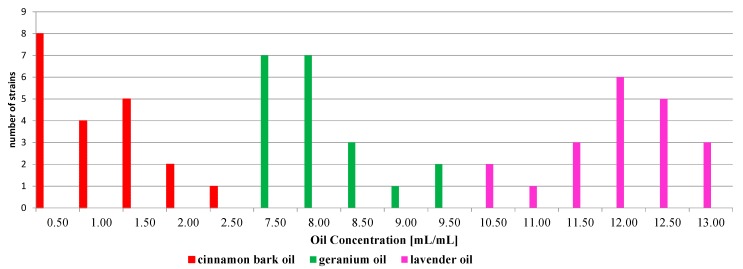
The susceptibility to oils of the *Acinetobacter baumannii* strains.

The MIC values for twenty tested *A. baumannii* strains ranged from 0.5 to 2.5 µL/mL. A concentration of 0.5 µL/mL inhibited the activity of standard strain *Acinetobacter baumannii* ATCC 19606 as well as the greatest number of tested strains (*n* = 8). The susceptibility to cinnamon bark, geranium and lavender oils of the *Acinetobacter baumannii* strains is presented in the Figure 2.

### 2.4. The Susceptibility of Acinetobacter Baumannii Bacterial Strains to Geranium Oil

The geranium oil was less active against *A. baumannii* strains isolated from the clinical materials, hospital equipment and environment. Fourteen of twenty tested strains were susceptible to geranium oil at concentrations between 7.5 and 8.0 µL/mL. The MIC value for *Acinetobacter baumannii* ATCC 19606 was 8.0 µL/mL.

### 2.5. The Susceptibility of Acinetobacter Baumannii Bacterial Strains to Lavender Oil

Of the tested essential oils, lavender oil demonstrated the weakest inhibitory effects against *Acinetobacter baumannii* strains. The MIC values were found to be between 10.5 and 13.0 µL/mL. The largest number of tested strains, *n* = 6 and *n* = 5, were inhibited at concentrations of 12.0 µL/mL and 12.5 µL/mL, respectively. The standard strain was susceptible to lavender oil at a concentration of 10.5 µL/mL.

### 2.6. The Effects of Essential Oils on the Viability of Human Microvascular Endothelial Cells (HMEC-1) and Glioblastoma Cell Line (T98G)

Cell viability was determined by the MTT assay. In general, a dose-dependent decrease in the survival of the two cell lines was observed. Of all the essential oils investigated, cinnamon bark oil exhibited the strongest cytotoxicity against HMEC-1 (IC_50_ = 0.0096 µL/mL), but T98G cells were less sensitive (IC_50_ = 0.322 µL/mL). 

**Figure 3:**
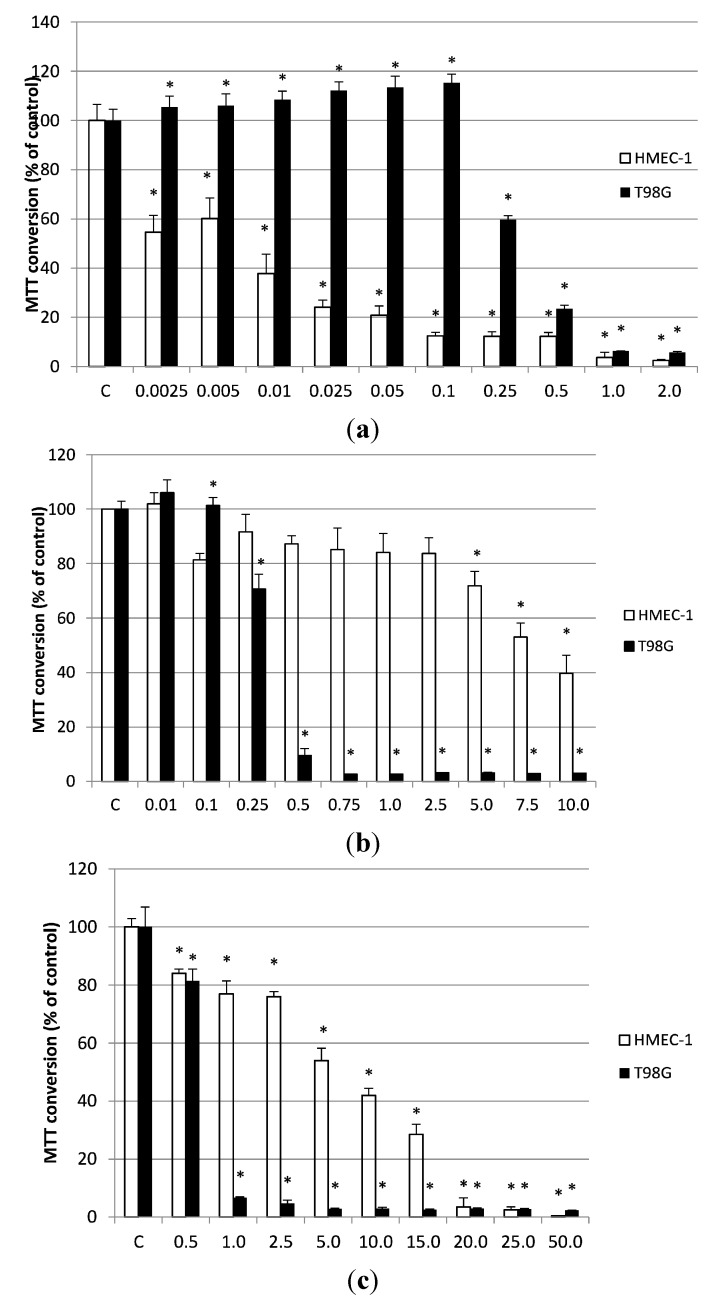
The effects of (**a**) cinnamon bark oil, (**b**) geranium oil and (**c**) lavender oil (24 h exposure) on the viability of human microvascular endothelial cells (HMEC-1) and glioblastoma cell line (T98). Bars represent the means (±SEM of 4–8 experiments). * *p* < 0.05 *vs.* control.

The geranium oil exhibited a similar cytotoxic activity against the T98G cell line as cinnamon bark oil (IC_50_ = 0.329 µL/mL) but the lowest cytotoxicity of the three oils against HMEC-1 (IC_50_ = 8.25 µL/mL). Finally, the lavender oil exhibited the lowest cytotoxicity towards T98 cells, with the IC_50_ values of lavender against HMEC-1 and T98G cells being 5.15 µL/mL and 2.27 µL/mL, respectively. The effects of all three essential oils on the viability of HMEC-1 and T98G are presented in Figure 3.

### 2.7. Discussion

The literature lacks detailed studies detailing the exact chemical composition of essential oils with antimicrobial effects and their activity against bacteria with a considerable degree of resistance to a wide range of recommended antibiotics. The essential oils tested in the present study were found to inhibit all tested *Acinetobacter baumannii* strains isolated from patients, hospital equipment and environment with a degree of resistance to antibiotics. The MIC values do not depend on the level of bacterial resistance.

Prakasam *at al*. report that clove, peppermint and eucalyptus oils possess antibacterial activity against clinical isolates of *A. baumannii* obtained from such varied clinical specimens as blood, endotracheal aspirates, bronchoalveolar lavage, pus, urine and sputum [19]. It should be noted that the tested bacterial strains were resistant to meropenem, which is the most effective drug for the treatment of infections caused by this notorious pathogen. In the present study, the tested *Acinetobacter baumannii* strains were characterised by similar resistance (50%) to the carbapenems meropenem and imipenem, and to such antibiotics as aztreonam, cefotaxim, ciprofloxacin, piperacillin and trimethoprim/sulfamethoxazole (100%).

This study has demonstrated that two essential oils, cinnamon bark and lavender oils, were cytotoxic to HMEC-1 at concentrations lower than the MIC. The geranium oil was less cytotoxic than lavender and cinnamon oils, with its IC_50_ value being close to that of the MIC. These results show that geranium oil may be safer towards HMEC-1 cells than the other oils. A different situation was observed in T98G cells, where the three essential oils were cytotoxic to cancer cells at concentrations which were lower than the MIC. Both the geranium and cinnamon oils were found to have similar cytotoxic activity against T98G cells, with the lavender oil exhibiting 2-fold weaker cytotoxic activity. Prashar *et al*. suggest that the cytotoxic mechanism of the oils may be associated with membrane damage [20].

However, the present findings suggest that while oils should be used in diluted forms, especially when directly applied to the skin, their antimicrobial properties are as effective as those of chemical antibacterial agents. In addition, it is important that the microorganisms do not acquire resistance to the essential oils or to their components [21]. Mayaud *et al*., proved high antibacterial activity of essential oil from *Cinnamomum verum* containing cinnamaldehyde (68.79%) and eugenol (6.96%) and from *Lavandula angustifolia* containing linalil acetate (37.68%) and linalool (26.57%) against Gram-positive and Gram-negative clinical strains. The MICs for antibiotic-resistant and antibiotic-susceptible strains were identical. The test clinical strains of *Acinetobacter baumannii* were susceptible to cinnamon bark oil at 0.08% ± 00% (v/v) and to lavender oil at 1.88% ± 0.63 (v/v) concentrations [22]. Moreover, the results obtained by Fani and Kohanteb showed a high inhibitory activity of *Cinnamon zeylanicum* oil against bacteria isolated from patient with oral infections. The MIC values for cinnamon oil were 12.8 μg/mL for multidrug resistant and non-multidrug resistant streptococci and 25.6 μg/mL for methicillin-resistant *Staphylococcus aureus* with the use of agar dilution method [23]. Pinto *et al*., evaluated the antibacterial activity of trans-cinnamaldehyde against nosocomial enteric bacilli producing β-lactamases by microdilution method [24]. The authors suggest that trans-cinnamaldehyde can be employed in solutions in the processes of disinfection of hospital instruments and equipment and in drug development for topical action. Our previous study showed that geranium oil containing citronellol (26.7%) and geraniol (13.4%) has the high antibacterial activity against resistant Gram-negative clinical strains of *E. coli*, *C. freundii*, *E. sakazakii*, *E. cloacae*, *P. mirabilis* and *P. aeruginosa* isolated from wound infections at concentrations from 3.0 µL/mL to 10.5 µL/mL [25]. Probuseenivasan *et al*., reported that geranium essential oil was active against Gram-negative standard strains as *E. coli*, *K. pneumoniae*, *P. aeruginosa*, *P. vulgaris* with MIC values between >6.4 and >12.8 mg/mL by agar dilution method. They also showed that cinnamon oil containing cinnamaldehyde (52.42%) was more active against tested bacteria–MIC values were between >0.8 and 3.2 mg/mL [7].

Essential oils have not only the ability to inhibit bacterial growth activity, but also to reduce the required active concentration of antibiotics by their synergistic activity. Guerra at al. found that essential oil from *Cinnamomum zeylanicum* and *Citrus limon* showed a synergistic effect in combinations with amikacin against *A. baumannii* strains [26]. According to patent authors, essential oils effectively prevent the accumulation of bacterial resistance to antibiotics, research on implementing them as an add-on anti-infective therapy should be performed [27,28]. It is widely known that chemical sanitizers are very often ineffective, and essential oils can be used for the decontamination of hard or soft surfaces, such as tables, cutting surfaces, bathroom fixtures, showers, tubs, sponges, shower curtains, plumbing fixtures and cutlery in the hospital environment.

## 3. Experimental Section

### 3.1. Bacterial Strains

The standard strain, *Acinetobacter baumanii* ATCC 19606, was acquired from the collection of the Medical and Sanitary Microbiology Department, Medical University of Lodz, Lodz, Poland. The tested *Acinetobacter baumannii* strains were obtained during 2009 and 2012 from a range of clinical materials and hospital equipment taken from a number of wards of a Medical University hospital in Lodz: surgery, anesthesiology, intensive care unit, cardiology and nephrology. The tested bacterial strains were isolated from the bronchia (*n* = 5), wounds (*n* = 3), urine (*n* = 2), catheters (*n* = 1), pus (*n* = 2), drain (*n* = 1), skin (*n* = 1), anus (*n* = 1), throat (*n* = 1), advanced tracheal tube (*n* = 1), bronchoscope (*n* = 1), equipment surface (*n* = 1).

### 3.2. Bacterial Strain Identification

*Acinetobacter baumannii* clinical strains were cultured on Columbia Agar (bioMerieux, Craponne, France) and on MacConkey Agar (bioMerieux). Microorganism species were identified using API 20 NE tests (bioMerieux). The bacteria were incubated in 37 °C for 24 h. *Acinetobacter baumannii* ATCC 19606 strain was used as a control.

### 3.3. Essential Oil Analysis

Commercial essential oils from bark of *Cinnamomum zeylanicum* Ness (*Lauraceae*), from the herb of *Pelargonium graveolens* Ait. (*Geraniaceae*) and from the flowering herb of *Lavandula angustifolia* Mill. (*Lamiaceae*) were purchased from the manufacturer (Pollena-Aroma, Warsaw, Poland) and analyzed by GC-FID-MS in the Institute of General Food Chemistry, Lodz University of Technology, Lodz, Poland, using a Trace GC Ultra apparatus (Thermo Fisher Scientific Inc., Waltham, MA, USA) MS DSQ II detectors and FID-MS splitter (SGE, Trajan Scientific Europe Ltd, Milton Keynes, UK). Identification of components was based on the comparison of their MS spectra with those of the laboratory-made MS library, commercial libraries (NIST 98.1, Wiley Registry of Mass Spectral Data, 8th Ed. and MassFinder 3.1 [29,30,31] and with literature data [32,33] along with the retention indices on apolar column (Rtx-1, MassFinder 3.1, Restek Corporation, Bellefonte, PA, USA) associated with a series of alkanes with linear interpolation (C_8_–C_26_).

### 3.4. Antibacterial Tests

Minimal Inhibitory Concentration (MIC) of Cinnamon, Geranium and Lavender Oils Assay

The MIC values were determined by the micro-dilution broth method. The essential oils were diluted in ethanol. This solutions were mixed with a 100 µL Mueller-Hinton broth to obtain concentrations from 0.25 to 3.0 µL/mL for cinnamon bark oil, and from 7.0 to 10.0 µL/mL for geranium oil and for lavender oil between 10.0 and 13.5 µL/mL. An inoculum containing 1.5 × 10^8^ CFU (10 µL) per well was added to broth with various oil concentrations to 96-well microtiter plates. The Minimal Inhibitory Concentration—MIC was determined after 24 h of incubation at 37 °C under aerobic conditions and estimated by visual inspection of the microplates. Control media containing only alcohol at concentrations used in the dilutions of oils did not inhibit the growth of bacterial strains.

### 3.5. Susceptibility Testing

The following antibiotics (Becton Dickinson, Franklin Lakes, NJ, USA) were used for susceptibility testing of *Acinetobacter baumannii* strains: AK-amikacin (30 μg), AMC-amoxicillin/clavulanic acid (20 µg/10 µg), ATM-aztreonam (30 μg), CN-gentamicin (30 μg), CT-colistin (50µg), CAZ-ceftazidim (30 μg), CTX-cefotaxim (30 µg), CIP-ciprofloxacin (5 μg), FEP-cefepim (30 μg), IPM-imipenem (10 μg), MEM-meropenem (10 μg), PLR-piperacillin (30 µg), SXT-trimethoprim/sulfamethoxazole (1.25/23.75 μg), TGC-tigecyclin (15 µg), TOB-tobramycin (10 μg), TZP-piperacillin/tazobactam (100/10 μg). Susceptibility testing was carried out using the disc-diffusion method, on Mueller-Hinton II Agar (bioMerieux). Cultures were incubated at 37 °C for 16–18 h. The results were interpreted according to EUCAST guidelines [34].

### 3.6. Cell Cultures

HMEC-1 (human microvascular endothelial cells) were purchased from ATCC (Rockville, MD, USA), catalog number ATCC-CRL-10636 (depositor Centers for Disease Control, Edwin W. Ades, Atlanta, GA, USA). For experimentation, cells between passages 10–31 were used. HMEC-1 cells were cultured in 25 cm^3^ flasks in MCDB 131 medium supplemented with 10% fetal bovine serum (Invitrogen, Carlsbad, CA, USA), 10 ng/mL epidermal growth factor, 1 μg/mL hydrocortisone and penicillin-streptomycin solution (Sigma-Aldrich Chemical Co. Ltd, St. Louis, MO, USA), in a humidified atmosphere of 95% and 5% CO_2_ at 37 °C. Cells were harvested every third day in a trypsin-EDTA solution (0.25% trypsin, 1 mM EDTA). HMEC-1 cells were cultured according to the method described in the literature [35,36] and the authors’ own modifications. T98G glioma cell line was purchased from American Type Culture Collection (ATCC). The cells were cultured in 25 mL flasks in medium composed of Advanced MEM supplemented with 10% fetal bovine serum, 2 mM glutamine and a penicillin-streptomycin solution, in a humidified atmosphere of 95% air and 5% CO_2_ at 37 °C. For subcultures, cells were harvested every third day in trypsin-EDTA (0.25% trypsin, 1 mM EDTA) solution.

### 3.7. MTT Conversion

The viability of the HMEC-1 (human microvascular endothelial cells) cells and the glioblastoma cell line (T98G) was measured using the 3-(4,5-dimethylthazol-2-yl)-2,5-diphenyltetrazolium bromide (MTT; Sigma-Aldrich Chemical Co. Ltd) conversion method. Cells were seeded (50,000 cells/well) into 96-well plates. The treated cells were incubated for 24 h with oils and without tested oils (control group). After incubation, 50 μL MTT (1 mg/mL, Sigma-Aldrich Chemical Co. Ltd, Saint Louis, MO, USA) was added and the plates were incubated at 37 °C for 3 h. At the end of the experiment, the cells were exposed to 100 μL dimethyl sulphoxide, which enabled the release of the blue reaction product: formazan. The absorbance at 570 nm was read on a microplate reader and results were expressed as a percentage of the absorbance measured in control cells.

### 3.8. Statistical Analysis

Statistical comparisons between the groups were performed using ANOVA, and post-hoc comparisons were performed using the Student-Newman-Keuls test. The normal distribution of parameters was checked by means of the Shapiro-Wilk test. If the data was not normally distributed or the values of the variance (test F) were different, ANOVA with Kruscal-Wallis and Mann-Whitney’s U test were used. All parameters were considered significantly different if *p* < 0.05. The statistical data analysis was performed using Statgraphics 5.0 plus software (STSC Inc., Rockville, MD, USA).

## 4. Conclusions

1. The obtained results show that the cinnamon, geranium and lavender essential oils demonstrate inhibitory activity against resistant clinical and environmental strains of *Acinetobacter baumannii.*
2. Cinnamon bark oil was the most active against all *Acinetobacter baumannii* bacteria.
3. Despite the fact that the bacteria are characterized by a high degree of antibiotic resistance, the tested essential oils have strong antibacterial action.

## References

[1] Maragakis L.L., Perl T.M. (2008). *Acinetobacter baumannii*: Epidemiology, Antimicrobial Resistance, and Treatment Options. Clin. Infect. Dis..

[2] Fournier P.E., Richet H., Weinstein R.A. (2006). The Epidemiology and Control of *Acinetobacter baumannii* in Health Care Facilities. Clin. Infect. Dis..

[3] Cusri S., Chongsuvivatwong V., Riviera J.I., Silpapojakul K., McNeil E., Doi Y. (2014). Clinical Outcomes of Hospital-Acquired Infection with *Acinetobacter nosocomialis* and *Acinetobacter pitii*. Antimicrob. Agents Chemother..

[4] Inouye S., Takizawa T., Yamaguchi H. (2001). Antibacterial activity of essential oils and their major constituents against respiratory tract pathogens by gaseous contact. J. Antimicrob. Chemother..

[5] Inouye S., Yamaguchi H., Takizawa T. (2001). Screening of the antibacterial effects of a variety of essential oils on respiratory tract pathogens, using a modified dilution assay method. J. Infect. Chemother..

[6] Sivamani P., Hameed A.S.S. (2010). *In vitro* antibacterial activity of essential oils of selected herbals against isolates from HIV/AIDS patients. J. Pharm. Res..

[7] Prabuseenivasan S., Jayakumar M., Ignacimuthu S. (2006). *In vitro* antibacterial activity of some plant essential oils. BMC Complement. Altern. Med..

[8] Cavanagh H.MA., Wilkinson J.M. (2005). Lavender essential oil: A review. Aust. Infect. Control..

[9] Hammer K.A., Carson C.F., Riley T.V. (1999). Antibacterial activity of essential oils and other plant extracts. J. Appl. Microbiol..

[10] Manosroi J., Dhumtanom P., Manosroi A. (2006). Antiproliferative activity of essential oil extracted from Thai medicinal plants on KB and P388 cell lines. Cancer Lett..

[11] Tsuneki H., Ma E.L., Kobayashi S., Sekizaki N., Maekawa K., Sasaoka T., Wang M.W., Kimura I. (2005). Antiangiogenic activity of beta-eudesmol *in vitro* and *in vivo*. Eur. J. Pharmacol..

[12] Guba R. (2001). Toxicity myths essential oils and their carcinogenic potential. Int. J. Aromather..

[13] Stammati A., Bonsi P., Zucco F., Moezelaar R., Alakomi H.I., von Wright A. (1999). Toxicity of selected plant volatiles in microbial and mammalian short-term assays. Food Chem. Toxicol..

[14] Lahlou S., Leal-Cardoso J., Duarte G. (2003). Antihypertensive effects of the essential oil of *Alpinia zerumbet* and its main constituent, terpinen-4-ol, in DOCA-salt hypertensive conscious rats. Fundam. Clin. Pharmacol..

[15] Dijoux N., Guingand Y., Bourgeois C., Durand S., Fromageot C., Combe C., Ferret P.J. (2006). Assessment of the phototoxic hazard of some essential oils using modified 3T3 neutral red uptake assay. Toxicol. In Vitro.

[16] Bleasel N., Tate B., Rademaker M. (2002). Allergic contact dermatitis following exposure to essential oils. Australas. J. Dermatol..

[17] (2008). European Pharmacopoeia,.

[18] (2008). Polish Pharmacopeia VIII.

[19] Prakasam G., Bhashini M., Lakshmipriya N., Ramesh S.S. (2014). *In-vitro* antibacterial activity of some essential oils against clinical isolates of *Acinetobacter baumannie*. Indian J. Med. Microbiol..

[20] Prashar A., Locke I.C., Evans C.S. (2004). Cytotoxicity of lavender oil and its major components to human skin cells. Cell Prolif..

[21] Yap P.S.X., Yiap B.C., Ping H.C., Lim S.H.E. (2014). Essential Oils, A New Horizon in Combating Bacterial Antibiotic Resistance. Open Microbiol. J..

[22] Mayaud L., Carricajo A., Zhiri A., Aubert G. (2008). Comparison of bacteriostatic and bactericidal activity of 13 essential oils against strains with varying sensitivity to antibiotics. Lett. Appl. Microbiol..

[23] Fani M.M., Kohanteb J. (2011). Inhibitory activity of *Cinnamon zeylanicum* and *Eucalyptus globulus* oils on *Streptococcus mutans*, *Staphylococcus aureus*, and *Candida* species isolated from patients with oral infections. Shiraz Univ. Dent. J..

[24] Pinto V., Barbosa C., Magalhães P., Coelho C., Fontenelle J., Cristino-Filho G., Chaves H., Silva A., Teixeira A., Bezerra M. (2014). Antimicrobial activity of the trans-cinnamaldehyde on nosocomial enteric bacilli producers of extended spectrum β-lactamase (ESBL). BMC Proc..

[25] Sienkiewicz M., Poznańska-Kurowska K., Kaszuba A., Kowalczyk E. (2013). The antibacterial activity of geranium oil against Gram-negative bacteria isolated from difficult-to-heal wounds. Burns.

[26] Guerra F.Q., Mendez J.M., Sousa J.P., Moralis-Braga M.F., Santos B.H., Melo Coutinho H.D., lima Ede O. (2012). Increasing antibiotic activity against a multidrug-resistant *Acinetobacter spp* by essential oils of *Citrus limon* and *Cinnamomum zeylanicum*. Nat. Prod. Res..

[27] Khanuja S.P.S., Srivastava S., Shasney A.K., Darokar M., Kumar T.R.S., Agarwal K.K., Ahmed A., Patra N.K., Sinha P., Dhawan S. (2004). Formulation Comprising Thymol Useful in the Treatment of Drug Resistant Bacterial Infections. U.S. Patent.

[28] Johnson E.A., Brehm-Stecher B.F. (2001). Method of Sensitizing Microbial Cells to Antimicrobial Compound. U.S. Patent.

[29] (1998). NIST 98.1 NIST/EPA/NIH Mass Spectral Library.

[30] (2008). Wiley Registry of Mass Spectral Data.

[31] (2007). MassFinder 3.1 Mass Spectral Library “Terpenoids and Related Constituents of Essential oils”.

[32] Adams R.P. (2007). Identification of Essential Oil Components by Gas Chromatography/Mass Spectroscopy.

[33] Joulain D., Konig W.A. (1998). The Atlas of Spectral Data of Sesquiterpene Hydrocarbons.

[34] European Committee on Antimicrobial Susceptibility Testing (EUCAST) (2012). Breakpoint Tables for Interpretation of MICs and Zone Diameters.

[35] Ades E.W., Candal F.J., Swerlick R.A., George V.G., Summers S., Bosse D.C., Lawley T.J. (1992). HMEC-1: Establishment of an immortalized human microvascular endothelial cell line. J. Investig. Dermatol..

[36] Wiktorowska-Owczarek A. (2014). The effect of diclofenac on proliferation and production of growth factors by endothelial cells (HMEC-1) under hypoxia and inflammatory conditions. Acta Pharm..

